# Progress Toward Global Eradication of Dracunculiasis, January 2016–June 2017

**DOI:** 10.15585/mmwr.mm6648a3

**Published:** 2017-12-08

**Authors:** Donald R. Hopkins, Ernesto Ruiz-Tiben, Mark L. Eberhard, Sharon L. Roy, Adam J. Weiss

**Affiliations:** ^1^The Carter Center, Atlanta, Georgia; ^2^Division of Parasitic Diseases and Malaria, Center for Global Health, World Health Organization Collaborating Center for Research, Training, and Eradication of Dracunculiasis, CDC.

Dracunculiasis (Guinea worm disease) is caused by *Dracunculus medinensis*, a parasitic worm. Approximately 1 year after a person acquires infection from contaminated drinking water, the worm emerges through the skin, usually on a lower limb (*1*). Pain and secondary bacterial infection can cause temporary or permanent disability that disrupts work and schooling. The campaign to eradicate dracunculiasis worldwide began in 1980 at CDC. In 1986, the World Health Assembly called for dracunculiasis elimination,* and the global Guinea Worm Eradication Program, led by the Carter Center and supported by the World Health Organization (WHO), United Nations Children's Fund, CDC, and other partners, began assisting ministries of health in countries with endemic dracunculiasis. In 1986, an estimated 3.5 million cases occurred each year in 20 countries in Africa and Asia (*2*). Since then, although the goal of eradicating dracunculiasis has not been achieved, considerable progress has been made. Compared with the 1986 estimate, the annual number of reported cases in 2016 has declined by >99%, and cases are confined to three countries with endemic disease. This report updates published (*3**–**4*) and unpublished surveillance data reported by ministries of health and describes progress toward dracunculiasis eradication during January 2016–June 2017. In 2016, a total of 25 cases were reported from three countries (Chad [16], South Sudan [six], Ethiopia [three]), compared with 22 cases reported from the same three countries and Mali in 2015 (Table 1). The 14% increase in cases from 2015 to 2016 was offset by the 25% reduction in number of countries with indigenous cases. During the first 6 months of 2017, the overall number of cases declined to eight, all in Chad, from 10 cases in three countries (Chad [four], South Sudan [four] and Ethiopia [two]) during the same period of 2016. Continued active surveillance, aggressive detection, and appropriate management of cases are essential eradication program components; however, epidemiologic challenges, civil unrest, and insecurity pose potential barriers to eradication.

**TABLE 1:** Number of reported indigenous human dracunculiasis cases, by country –– worldwide, January 2015–June 2017

Country	Cases by period					
	Jan–Dec 2015	Jan–Dec 2016	% Change Jan–Dec 2015 to Jan–Dec 2016	Jan–Jun, 2016*	Jan–Jun, 2017	% Change Jan–Jun 2016 to Jan–Jun 2017
	No.	No. (% contained)		No.	No. (% contained)	
Chad	9	16 (56)	78	4	8 (75)	100
Ethiopia	3	3 (67)	0	2	0 (—)	**-**100
Mali^†^	5	0 (—)	**-**100	0	0 (—)	0
South Sudan	5	6 (50)	20	4	0 (—)	**-**100
**Total**	**22**	**25 (56)**	**14**	**10**	**8 (75)**	**-20**

* No international importations were reported during the 18-month period January 2016–June 2017.

^†^ Civil unrest and insecurity continued to constrain program operations in regions with endemic dracunculiasis (Gao, Kidal, Mopti, and Timbuktu) during 2016–2017.

Because the life cycle of *D. medinensis* is complex, its transmission can be interrupted using multiple strategies (*1*). Dracunculiasis can be prevented by the following four main interventions: 1) educating residents in communities where the disease is endemic, particularly persons from whom worms are emerging, to avoid immersing affected body parts in sources of drinking water; 2) filtering potentially contaminated drinking water through a cloth filter or pipe filters to remove copepods (small crustaceans that host *D. medinensis* larvae); 3) treating potentially contaminated surface water with the organophosphate insecticide temephos (Abate) to kill the copepods; and 4) providing safe drinking water from bore-hole or protected hand-dug wells (*5*). Containment^†^ of transmission is achieved through four complementary measures: 1) voluntary isolation and education of each patient to prevent contamination of drinking water sources, 2) provision of first aid to prevent secondary infections, 3) manual extraction of the worm, and 4) application of occlusive bandages. No vaccine or medicine to prevent or treat Guinea worm disease currently exists.

*D. medinensis* has an approximately 1-year incubation period (range = 10–14 months) after infection (*5*). A case of dracunculiasis is defined as an infection occurring in a person exhibiting a skin lesion or lesions with emergence of one or more worms that are laboratory-confirmed at CDC as *D. medinensis*. Each infected person is counted as a case only once during a calendar year. Because certain patients have multiple Guinea worms emerge, more laboratory-confirmed specimens than cases might be reported in any given period.

Countries enter the WHO precertification stage of eradication after 1 full year with no reported indigenous^§^ cases. An imported case is an infection resulting from ingestion of contaminated water from a source, identified through patient interviews and epidemiologic investigation, in a place other than in the community where the patient is detected and the case reported (i.e., another country or village within the same country). Since 2012, no known internationally imported cases have been reported.

In each affected country, a national dracunculiasis eradication program receives monthly reports regarding cases from each village under active surveillance. Reporting rates are calculated as the proportion of all villages under active surveillance reporting monthly (Table 2). Active surveillance is conducted in all villages with endemic dracunculiasis or that are at high risk for importation, with daily searches of households for persons with signs or symptoms of dracunculiasis, to ensure case detection within 24 hours of worm emergence and prompt patient management to prevent contamination of water sources. Villages where endemic transmission of dracunculiasis is interrupted (i.e., zero cases reported for ≥12 consecutive months) are kept under active surveillance for 3 consecutive years. WHO certifies a country free from dracunculiasis after that country maintains adequate nationwide surveillance for ≥3 consecutive years and demonstrates that no indigenous cases occurred during that period. As of January 2016, WHO had certified 198 countries, areas, and territories as free from dracunculiasis (*3*). Eight countries remain to be certified: four where dracunculiasis is currently endemic (Chad, Ethiopia, Mali, and South Sudan), two in the precertification stage (Kenya and Sudan), and two never known to have had endemic dracunculiasis since the global eradication program began in 1980 (Angola and the Democratic Republic of the Congo), which are in the process of completing the requirements for certification.

**TABLE 2:** Reported human dracunculiasis cases, surveillance, and status of local interventions in villages with endemic disease, by country –– worldwide, 2016

Cases/Surveillance/Intervention status	Country				
	Chad*	Ethiopia	Mali^†^	South Sudan	Total
**Reported cases**					
No. indigenous, 2016	16	3	0	6	**25**
No. imported,^§^ 2016	0	0	0	0	**0**
% Contained^¶^ in 2016	56	67	0	50	**56**
% Change in indigenous cases in villages/localities under surveillance, same period 2015 and 2016	78	0	**-**100	20	**14**
**Villages under active surveillance, 2016**					
No. of villages	1,799	152	450	2,736	**5,137**
% Reporting monthly	100	89	100	99	**99**
No. reporting ≥1 case	8	3	0	5	**16**
No. reporting only imported** cases	0	0	0	0	**0**
No. reporting indigenous cases	8	3	0	5	**16**
**Status of interventions in villages with endemic dracunculiasis, 2015–2016**					
No. of villages with endemic dracunculiasis	20	5	3	9	**37**
% Reporting monthly^††^	100	100	100	100	**100**
% Filters in all households^††^	100	100	100	100	**100**
% Using temephos^††^	30	100	100	100	**60**
% ≥1 safe water source^††^	73	100	66	56	**62**
% Providing health education^††^	100	100	100	100	**100**

* Participants at the annual Chad Guinea Worm Eradication Program review meeting in November 2014 adopted “1+ case village” as a new description for villages in Chad affected by human cases of Guinea worm disease or dogs infected with Guinea worms and defined as “a village with one or more indigenous or imported cases of Guinea worm infections in humans, dogs, or cats in the current calendar year or previous year.”

^†^ Civil unrest and insecurity continued to constrain Guinea Worm Eradication Program operations (supervision, surveillance, and interventions in Gao, Kidal, and Timbuktu regions).

^§^ Imported from another country.

^¶^ Transmission from a patient with dracunculiasis is contained only if all of the following conditions are met for each emerged worm: 1) the infected patient is identified ≤24 hours after worm emergence; 2) the patient has not entered any water source because the worm emerged; 3) a village volunteer or other health care provider has managed the patient properly, by cleaning and bandaging the lesion until the worm has been fully removed manually and by providing health education to discourage the patient from contaminating any water source (if two or more emerging worms are present, transmission is not contained until the last worm is removed); 4) the containment process, including verification of dracunculiasis, is validated by a Guinea Worm Eradication Program supervisor within 7 days of emergence of the worm; and 5) temephos is used to treat potentially contaminated surface water if any uncertainty about contamination of these sources of drinking water exists, or if a such a source of drinking water is known to have been contaminated.

** Imported from another in-country village with endemic disease.

^††^ The denominator is the number of endemic villages/localities where the program applied interventions during 2015–2016.

During January 2016–June 2017, CDC evaluated 118 worm specimens that emerged from humans, including 108 (91.5%) from the four countries with endemic dracunculiasis, two (1.7%) from Kenya, three (2.5%) from Benin (Kenya and Benin formerly had endemic dracunculiasis), four (3.4%) from the Democratic Republic of Congo, and one (1%) from Niger. Among the 118 human worm specimens submitted, 89 (75%) were from 2016 (37 [42%] were identified as *D. medinensis*) and 29 were from January to June 2017 (eight [28%] were identified as *D. medinensis*).

During 2016, 46 animal worm specimens were submitted, and 32 (70%) were identified as *D. medinensis*. The 32 *Dracunculus* specimens came from two baboons and 13 dogs from Ethiopia, 11 dogs from Mali, and five dogs and one domestic cat from Chad. During January–June 2017, 18 animal worm specimens were submitted and 14 were identified as *D. medinensis*. The 114 *Dracunculus* specimens came from four baboons and nine dogs from Ethiopia, and one dog from Chad.

## Country Reports

**Chad.** After a decade with no reported cases, Chad reported 10 indigenous cases in 2010. After indigenous cases were confirmed during 3 consecutive years, dracunculiasis was declared to be endemic in 2012 (*6*,*7*). In 2016, Chad reported 16 cases (nine contained) in 12 villages, compared with nine cases (none contained) in 2015. During the first half of 2017, eight cases (six contained) were reported in eight villages. One of 12 villages that reported a case in 2016, and one of eight reporting a case during January–June 2017, had reported a case previously.

In 2012, Guinea worm infections were first reported in domestic dogs in Chad (*6*), and since then, more dogs than humans have been identified with emerging Guinea worms. This substantial number of nonhuman infections has not occurred in any other country during the eradication campaign. Worm specimens obtained from dogs were determined to be genetically indistinguishable from *D. medinensis* worms removed from humans in Chad (*6*). A majority of infections during the current outbreak have occurred in communities along the Chari River. The Carter Center has assisted the ministry of health in implementing active village-based surveillance for the disease in approximately 1,700 villages in the at-risk zone. The working hypothesis, on the basis of biologic, environmental, and epidemiologic investigations by CDC and the Carter Center, is that the cases in humans and infected dogs are associated with the domestic and commercial fishing industry along the Chari River and involve fish, frogs, or other aquatic hosts that serve as paratenic hosts (intermediate hosts in which no development of the parasite occurs). New infections are thought to occur when humans consume inadequately cooked paratenic hosts and when such hosts are consumed raw by dogs (*6*). Overall, 1,011 infected dogs (and 11 infected domestic cats) were reported during 2016, which was twice the number of infected dogs (503) reported in 2015. However, during January–June 2017, 537 infected dogs were reported, which is an 18% decrease from the 653 reported during the same period of 2016. This is the first such half-yearly reduction since infected dogs were first reported in 2012, and it reflects consecutive months of declining dog infections that began in November 2016 (*3*).

Beginning in October 2013, Chad's Guinea Worm Eradication Program urged villagers to cook their fish well, bury fish entrails, and prevent dogs from eating fish entrails. By June 2017, according to monthly sample surveys, this intervention was being implemented by approximately 81% of respondents in surveyed communities with populations at risk. In February 2014, health education measures began to persuade villagers to tether infected dogs until the worms emerged, to prevent contamination of water and infection of copepods. In February 2015, the program introduced a reward equivalent to US$20 for reporting and tethering an infected dog. Whereas 40%, 68%, and 68% of infected dogs were tethered in 2014, 2015, and 2016, respectively, 78% of 537 infected dogs reported during January–June 2017 were tethered.

Beginning before 2010, Chad has offered a cash reward equivalent to US$100 for reporting a human case of dracunculiasis. In areas under active surveillance, 69% of 383 residents surveyed during January–June 2017 knew of the cash reward for reporting a case of dracunculiasis, and 60% of 363 persons surveyed knew of the cash reward for reporting and tethering an infected dog.

As of June 2017, 68% of villages with endemic dracunculiasis had safe water (i.e., water sources free of copepods, such as rapidly flowing rivers, protected hand dug wells, and borehole wells). Temephos use is limited by the extremely large lagoons used for fishing and as sources of drinking water. Starting in August 2014, an innovative technique of applying temephos to smaller cordoned sections of the lagoons at entry points used by infected humans or dogs was introduced and used to protect 19, 29, 61, and 51 villages in 2014, 2015, 2016, and January–June 2017, respectively.

The Carter Center and WHO Collaborating Center for Dracunculiasis Eradication at CDC are supporting research to better understand the unusual epidemiology of the current outbreak of dracunculiasis in Chad, assess antihelminthic treatment of dogs to prevent maturation of worms, and study the food sources and movements of dogs in an area of Chad with endemic disease. In collaboration with researchers from the University of Georgia, this initiative has demonstrated that *D. medinensis* can use an amphibian (frog) (*8*) as a paratenic host in the laboratory (*8*) and has recovered, for the first time, a *Dracunculus* larva from a frog captured in the wild in Chad (*9*).

**Ethiopia**. In 2016, Ethiopia reported three cases of dracunculiasis (two contained), in two villages of the Gog district and one village of the Lare district of Gambella Region. This is the same number of cases that Ethiopia reported in 2015 and in 2014. (The origin of the third case, which was reported in September 2016, is unclear, because the patient entered Ethiopia from South Sudan approximately 1 year before emergence of his Guinea worm). Ethiopia also reported 14 infected domestic dogs and two infected baboons in 2016, compared with 13 infected dogs and one infected baboon in 2015, all in the same area of the Gog district. During January–June 2017, Ethiopia reported no human case, eight infected dogs, and four infected baboons. However, in the same area of the Gog district, there were two cases in humans, two infected dogs, and no infected baboons during the same period of 2016. The program applied temephos monthly to almost all water sources known to have been used by humans in the at-risk area throughout 2015, increased coverage threefold to include numerous smaller water sources in 2016, and is addressing additional gaps in identification of water sources related to a particular stream in the at-risk area in 2017. A cash reward, equivalent to US$20 for reporting an infected animal was introduced, and the ministry of health held three press conferences to publicize the eradication effort during 2016. There are 152 villages under active surveillance in three districts of Gambella Region. Ethiopia offers a cash reward equivalent to US$100 for reporting a case of dracunculiasis. Among 11,712 persons surveyed in the Gog district during January–June 2017, 82% were aware of the reward for reporting an infected person; 61% of 2,123 surveyed knew of the reward for reporting an infected animal.

**Mali**. In 2016, Mali reported no human cases of dracunculiasis for the first time since its eradication program began, compared with five cases reported from three villages in 2015. Mali reported one infected dog for the first time in 2015 and 11 infected dogs (eight contained) in 2016. Two infected dogs (one contained) were reported during January–June 2017, compared with one dog (contained), during the same period of 2016. All infected dogs were detected in the Tominian district of Segou Region, but many had been imported from other areas of Segou or adjacent Mopti Region, in which certain areas were inaccessible to the program because of insecurity. Mali has 455 villages under active surveillance. Mali offers a cash reward equivalent to US$100 for reporting a case of dracunculiasis and US$20 for reporting and tethering an infected dog. In areas under active surveillance, 79% of 23,943 persons surveyed in 2016 were aware of the cash reward for reporting a case. During January–June 2017, 80% of 2,190 persons surveyed were aware of the reward for reporting a case, and 88% of 819 persons surveyed were aware of the reward for reporting and tethering an infected dog.

**South Sudan**. South Sudan reported six cases of dracunculiasis (three contained) from four villages in 2016, all west of the Nile, compared with five cases (three contained) reported from five villages in 2015. The country reported no infected dogs in 2016, compared with one in 2015, which has been the only infected dog found in South Sudan to date. South Sudan has reported no cases during January–June 2017, compared with four cases (three contained) reported during the same period of 2016. South Sudan has 3,860 villages under active surveillance. In April 2014, South Sudan began offering a cash reward equivalent to approximately US$125 for reporting a case of dracunculiasis (*10*). The overall level of reward awareness among 495 persons queried in active surveillance areas in 2016 was 76%. In March 2017, the ministry of health doubled its cash reward for reporting a case of dracunculiasis to 10,000 South Sudanese pounds (approximately US$139) to adjust for inflation and introduced a cash reward (approximately US$20) for reporting and tethering an infected animal. Coverage with interventions in villages with endemic disease remains high (except for provision of safe sources of drinking water) (Table 2), despite increased insecurity having forced the evacuation of most expatriate staff members assisting the South Sudan Guinea Worm Eradication Program since early July 2016.

## Discussion

The number of countries reporting endemic dracunculiasis decreased from four in 2015 to three in 2016, to only one country (Chad), which reported eight cases during January–June 2017. This compares with 10 cases reported by Chad, Ethiopia, and South Sudan during January–June 2016 and indicates that the goal of complete eradication of the disease is closer. The decrease in the number of infected dogs in Chad for the first time during January–June 2017 is another favorable milestone. Led by their ministers of health, Mali and Chad have launched enhanced national communication campaigns in March and July 2017, respectively, to increase awareness of rewards for reporting cases and knowledge of prevention messages; South Sudan and Ethiopia plan to launch similar campaigns later in 2017.

Political support for Guinea worm eradication remains strong in South Sudan and has improved recently in Chad, Ethiopia, and Mali. The health ministers of all four countries attended or were represented at the annual informal meeting of countries with current or former endemic dracunculiasis at the World Health Assembly in Geneva, Switzerland, in May 2016, and at the International Review Meeting for Guinea Worm Eradication Program Managers held at The Carter Center in March 2016. Mali’s Minister of Health visited an area with endemic disease in June 2016, whereas South Sudan’s Minster of Health and the regional Vice President of Gambella, Ethiopia, visited such areas in their countries in September 2016. In June 2017, Chad’s National Assembly convened a special session for a briefing on that country’s Guinea Worm Eradication Program. Insecurity remains a serious challenge to program activities, especially in Mali and South Sudan.

Additional interventions, including increased use of temephos and trials of potential anthelminthic treatments for infected dogs are beginning or underway in Chad. In addition, scientists are researching aspects of the parasite’s biology, life cycle, and molecular composition, and dog ecology. Furthermore, a case-control study of humans and dogs in households with and without infected dogs is planned in Chad for early 2018.

**Summary**

**What is already known about this topic?**

The number of new cases of dracunculiasis (Guinea worm disease) occurring worldwide has decreased from an estimated 3.5 million in 1986, when the World Health Assembly declared global elimination as a goal, to 25 in 2016.

**What is added by this report?**

The number of human dracunculiasis cases reported worldwide during 2016 increased to 25 cases in three countries in 2016 from 22 cases in four countries in 2015. However, during January–June 2017, the number of cases reported decreased from 10 cases in three countries during the same period in 2016, to eight, all in Chad. The number of infected domestic dogs doubled from 503 in 2015 to 1,011 in 2016, but declined to 537 during January–June 2017 compared with 653 during the same period of 2016. The emergence of infected dogs in Chad especially, and program disruptions caused by civil unrest and insecurity in Mali and South Sudan, are now the greatest challenges to interrupting transmission.

**What are the implications for public health practice?**

Although earlier target dates for global dracunculiasis eradication were missed, progress towards this goal has accelerated. However, rigorous implementation (including recent interventions to prevent transmission to and from dogs) must be maintained in Chad to ensure continued progress during 2017 and beyond.

## Footnotes

* http://www.who.int/neglected_diseases/mediacentre/WHA_39.21_Eng.pdf.

† Transmission from a patient with dracunculiasis is contained only if all of the following conditions are met for each emerged worm: 1) the infected patient is identified ≤24 hours after worm emergence; 2) the patient has not entered any water source because the worm emerged; 3) a village volunteer or other health care provider has managed the patient properly, by cleaning and bandaging the lesion until the worm has been fully removed manually and by providing health education to discourage the patient from contaminating any water source (if two or more emerging worms are present, transmission is not contained until the last worm is removed); 4) the containment process, including verification of dracunculiasis, is validated by a Guinea Worm Eradication Program supervisor within 7 days of emergence of the worm; and 5) temephos is used to treat potentially contaminated surface water if any uncertainty about contamination of these sources of drinking water exists, or if such a source of drinking water is known to have been contaminated.

§ An indigenous case of dracunculiasis is defined as an infection consisting of a skin lesion or lesions with emergence of one or more Guinea worms in a person who had no history of travel outside their residential locality during the preceding year.
