# *Erratum*: Vol. 66, No. 48

**DOI:** 10.15585/mmwr.mm6702a7

**Published:** 2018-01-19

In the report “Progress Toward Global Eradication of Dracunculiasis, January 2016–June 2017,” on page 1328, the last sentence of the third paragraph should have read “The **14** *Dracunculus* specimens came from four baboons and nine dogs from Ethiopia and one dog from Chad.”

On page 1331, under “Discussion,” the first sentence of the second paragraph should have read “Additional interventions, including increased use of temephos and trials of potential **antihelminthic** treatments for infected dogs, are beginning or underway in Chad.”

